# Strategies for calculating contrast media dose for chest CT

**DOI:** 10.1186/s41747-023-00345-w

**Published:** 2023-06-12

**Authors:** Mette Karen Henning, Catherine Gunn, Juan Arenas-Jiménez, Safora Johansen

**Affiliations:** 1 Faculty of Health Sciences, Department of Life Sciences and Health, Oslo Metropolitan University, Oslo, Norway; 2 Department of Radiology and Nuclear Medicine, Oslo University Hospital, Oslo, Norway; 3 School of Health Sciences, Dalhousie University, Halifax, Canada; 4 Department of Radiology, Dr. Balmis General University Hospital, Alicante, Spain; 5 Department of Pathology and Surgery, Miguel Hernández University, Alicante, Spain; 6 Alicante Institute for Health and Biomedical Research (ISABIAL), Alicante, Spain; 7 Department of Cancer Treatment, Oslo University Hospital, Oslo, Norway

**Keywords:** Body composition, Contrast media, Lean body weight, Thorax, Tomography (x-ray computed)

## Abstract

**Background:**

Total body weight (TBW) is a frequently used contrast media (CM) strategy for dose calculation in enhanced CT, yet it is suboptimal as it lacks consideration of patient characteristics, such as body fat percentage (BFP) and muscle mass. Alternative CM dosage strategies are suggested by the literature. Our objectives were to analyze the CM dose impact when adjusting to body composition using methods of obtaining lean body mass (LBM) and body surface area (BSA) along with its correlation with demographic factors in contrast enhanced chest CT examinations.

**Methods:**

Eighty-nine adult patients referred for CM thoracic CT were retrospectively included, categorized as either normal, muscular, or overweight. Patient body composition data was used to calculate the CM dose according to LBM or BSA. LBM was calculated with the James method, Boer method, and bioelectric impedance (BIA). BSA was calculated using the Mostellar formula. We then correlated the corresponding CM doses with demographic factors.

**Results:**

BIA demonstrated the highest and lowest calculated CM dose in muscular and overweight groups respectively, compared to other strategies. For the normal group, the lowest calculated CM dose was achieved using TBW. The calculated CM dose was more closely correlated with BFP using the BIA method.

**Conclusions:**

The BIA method is more adaptive to variations in patient body habitus especially in muscular and overweight patients and is most closely correlated to patient demographics. This study could support utilizing the BIA method for calculating LBM for a body-tailored CM dose protocol for enhanced chest CT examinations.

**Relevance statement:**

The BIA-based method is adaptive to variations in body habitus especially in muscular and overweight patients and is closely correlated to patient demographics for contrast-enhanced chest CT.

**Key points:**

• Calculations based on BIA showed the largest variation in CM dose.

• Lean body weight using BIA demonstrated the strongest correlation to patient demographics.

• Lean body weight BIA protocol may be considered for CM dosing in chest CT.

**Graphical Abstract:**

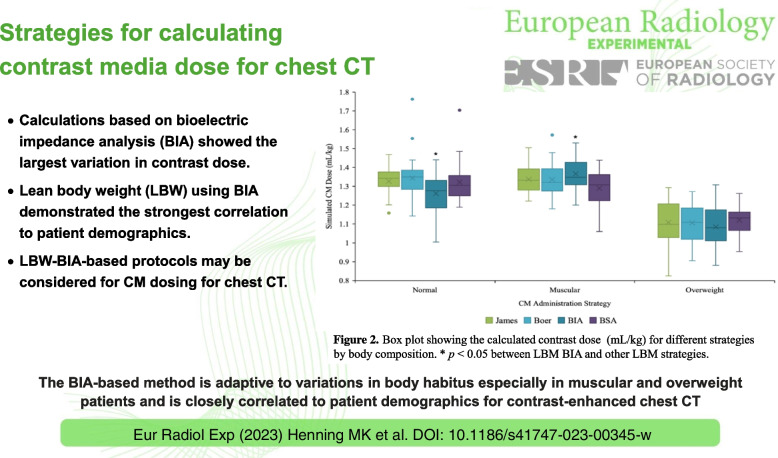

## Background

Image quality and lesion detection in computed tomography (CT) scans of the chest may be improved with the use of contrast media (CM). Traditionally, enhanced CT protocols have used fixed CM doses [1]; however, personalized CM approaches have been shown to be nearly as effective as personalized approaches to patient care [2, 3]. While iodine concentration, injection rate, scan delay, blood pressure and cardiac function can affect contrast enhancement, so too can body composition, which impacts distribution of CM in extracellular spaces due to differences in blood volume and flow [4, 5]. As a result, new strategies have emerged aiming to customize CM dose to both total body weight (TBW) [1, 6] and body composition [7]. Linear weight-based approaches, however, do not accurately estimate ideal enhancement as they fail to take into consideration patient characteristics, such as body fat percentage (BFP) and muscle mass [6, 8].

Literature documents numerous methods to customize CM doses, however none are recognized as standard or preferred [1, 8]. Lean body mass (LBM), defined as the difference between TBW and body fat weight, is suggested as one appropriate CM administration strategy [9]. CM dose based on LBM decreases CM doses while consistently improving image quality, as it corrects for high body fat percentage (BFP) by correlating with extracellular fluid volumes [10]. LBM can be calculated using the James method, the Boer method, and bioelectric impedance analysis (BIA) [6, 9, 11]. For obese patients, the Boer formula is more consistent and reliable [7] as the James method underestimates CM doses in this population and is therefore not appropriate in patients with body mass index (BMI) above approximately 40 kg/m^2^ [6, 9, 11]. BIA is considered the standard for evaluating body composition and BFP, measuring conduction of an electrical current through intracellular and extracellular water [6, 12]; however, it relies on constant patient hydration and may be inaccurate if a patient is NPO (Nothing by Mouth) prior to scanning [12]. Iodine dose may also be adjusted by measuring body surface area (BSA) [1], and when compared to LBM methods, it may be highly applicable clinically to adjust CM dose across a range of body sizes and weights [1]. Research shows that BSA calculated using the Mosteller formula is a strong indicator for metabolic mass [1]. However, controversy exists regarding BSA calculations as using actual weight-based calculations compared to ideal TBW may overestimate CM dose by 15–30% [13].

With a global shortage of iodinated CM in recent years [14], it is necessary to modify thinking with respect to how CM can most effectively be utilized [15–17]. Consequently, there is an increased focus on the reduction and personalization of CM doses to ensure adequate image quality. Current research on patient-tailored CM dosage focuses mostly on abdominal protocols [1, 4, 9, 18] without examining the impact of these methods in evaluation of the chest. In this setting, we aimed to investigate the impact of body composition data on various CM dose calculations.

## Methods

### Population

The present study uses patient data from a larger prospective study evaluating the CM dose used in contrast enhanced thoracic CT examinations. The larger study was approved by the Norwegian Regional Medical Research Ethics Committee (No. 2016/674) and Data Protection Officer at the institution. Informed consent was waived from patients referred for various chest CT examinations. The patients were randomized to receive CM according to either fixed or weight/body composition tailored CM approach. For the current study, the dataset from only weight/body composition tailored CM approach was used and the patient dataset was prospectively collected between 2019 and 2021 at a University Hospital in Norway for the CM dose calculation. All CT chest examinations were performed on the same scanner and had identical patient positioning, scan delay and CM was Iohexol 350 mgI/mL (Omnipaque, GE Healthcare, Oslo, Norway). The injection duration was 18 s, and consequently, the flow rate was manually adjusted individually for the included patients. CM injection was followed by a 40-mL saline flush at the same flow rate. Exclusion criteria were hemodynamic instability, cardiac failure, pacemaker, renal insufficiency (estimated glomerular filtration rate < 30 mL/min/1.73 m^2^), contraindications to CM, and age < 18 years. As this dataset was only utilized to calculate potential CM doses for TBW, LBM, and BSA approaches, no evaluation of image quality was performed.

**Fig. 1:**
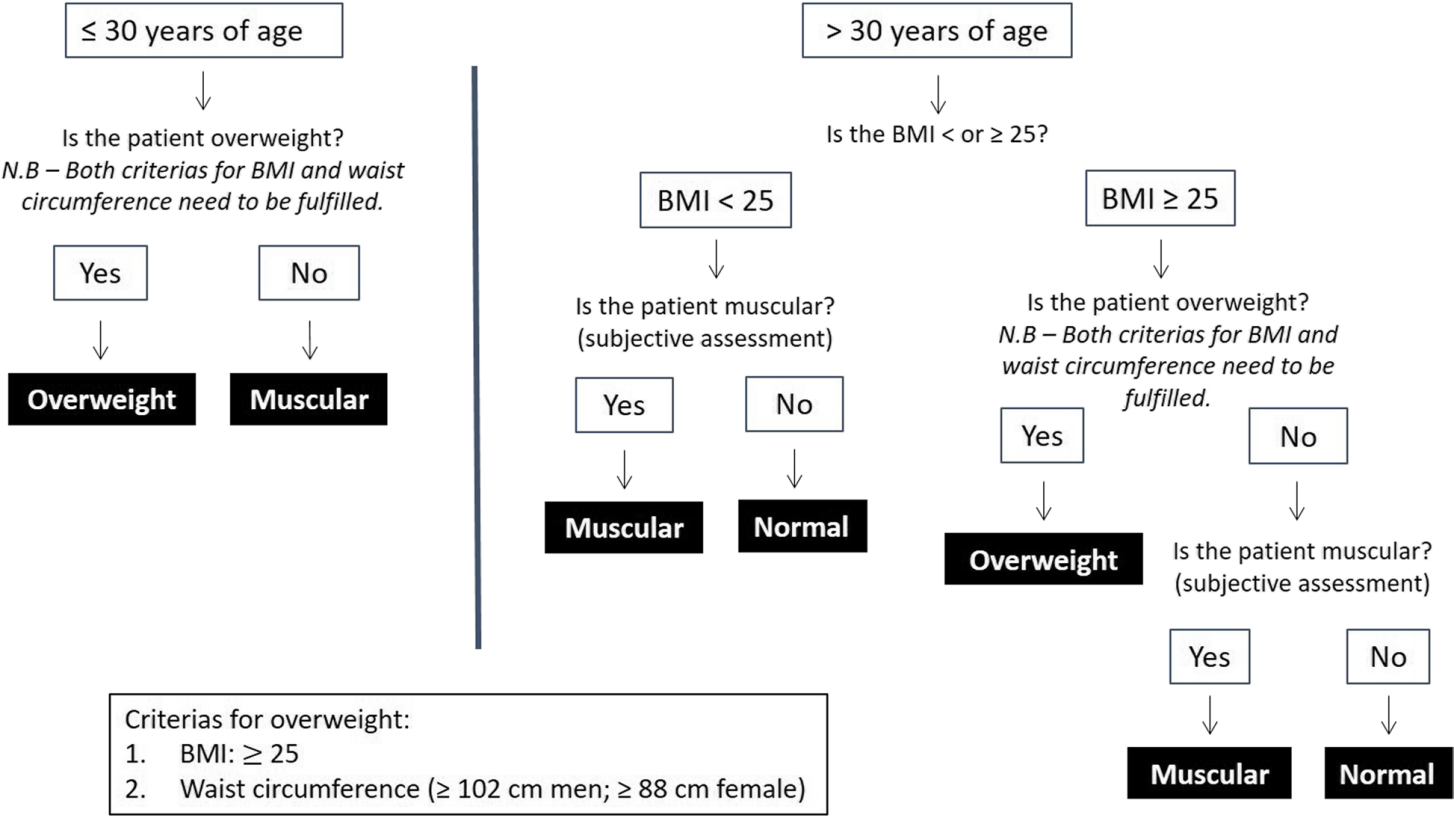
Schematic illustration of the selected criteria for determining body composition. *BMI*, Body mass index

Eighty-nine patients were included and categorized as either normal, muscular or overweight based on pre-defined objective and subjective criteria (Fig. 1). Criteria for inclusion in the normal group was a BMI < 25. Participants 30-years-old and younger with a BMI < 25 and waist circumference smaller than the overweight category were included in the muscular group. For patients > 30 years, a subjective assessment was performed to determine if they belonged to the muscular or other two groups. Participants were included in the overweight category if their BMI was ≥ 25 with a waist circumference ≥ 88 cm for females and ≥ 102 cm for males. The assessment was performed by a restricted number of specialized CT technologists/radiographers with experience and training to perform subjective assessment for the study [19].

Participants' age, TBW, height, BMI, waist circumferences, and BFP were recorded. The BFP was measured using a foot-to-foot bioelectrical impedance analyzer (BIA) (Tanita® mod. DC-430MA, Tanita Corp., Tokyo, Japan). The test took approximately 2−3 min, including entering the information into the software and generating the report.

### Patient allometric parameters

Patient allometric parameters were calculated using the formulas in Table 1. LBM reflects the central blood volume, extracellular fluid space of well-perfused tissues, and the small volume of the extracellular fluid space of poorly perfused tissues, such as the skeletal system [20]. LBM calculations using the James and Boer formulas estimate CM dose by considering the patient’s height, weight, and gender, unlike traditional TBW protocols [11]. Calculating LBM using the BIA formula accounts for BFP rather than height and does not differentiate between gender [12]. BSA (m^2^) was calculated using the Mosteller formula, which estimates body surface using height and weight [1] and is also independent of gender. Used often in clinical practice to correct drug dosage based on body size and as an alternative to the LBM methods, it is easily memorized and can be calculated on a handheld calculator [1, 20].

**Table 1:** Equations for calculating allometric parameters using three different LBM and BSA contrast medium strategies

Allometric parameters	Equation
LBM (kg)	
According to James	
*Female*	(1.07 • TBW) − 148 (TBW/H)^2^
*Male*	(1.10 • TBW) − 128 (TBW/H)^2^
According to Boer	
*Female*	(0.252 • TBW) + (0.473 • H) − 48.3
*Male*	(0.407 • TBW) + (0.267 • H) − 19.2
According to BIA	
*Female/male*	TBW (1–BFP/100)
BSA (m^2^)	
According to Mostellar formula	
*Female*/*male*	[(H • TBW)/3600]^0.5^

*LBM* Lean body mass, *BSA* Body surface area, *BIA* Bioelectric impedance analyzer, *TBW* Total body weight, *H* Height, *BFP* Body fat percentage

### CM dose calculations

Calculation of CM doses based on alternative allometric parameters described in Table 1 were achieved using the CM dose administered in mL to patients from the original data set. The CM dose was initially determined based on findings in a survey study, where CM dosages in chest CT ranged from 0.6 to 2.0 mL/kg with a mean and median CM dose of 1.2 mL/kg and 1.3 mL/kg TBW, respectively [21]. The total amount of CM dose (8,560 mL) received by the 89 included patients in the earlier larger prospective study, but this time independent of their body composition, was divided by TBW (6,967 kg), LBM James (5,064 kg), LBM Boer (5,067 kg), LBM BIA (4,997 kg) and BSA (173 m^2^) based on equations shown in Table 1. This resulted in 1.2 mL/kg using TBW, 1.7 mL/kg using LBM for the three LBM methods, and 49.0 mL/m^2^ for BSA. These resultant calculations were further used to calculate the CM dose based on the three included CM dose calculation strategies in the present retrospective study.

### Statistical analysis

Descriptive statistics for CM doses are expressed as means ± standard deviations with ranges. Normality was tested using graphical assessment together with the Shapiro–Wilk test. Using Pearson’s correlation coefficient *r*, correlations between calculated CM doses that patients would have received for each CM strategy and their TBW, height, BMI, waist circumference, and BFP, were calculated overall and separately for each body composition. Correlations were considered weak (*r*, -0.00 to 0.40 or 0.00 to -0.40), moderate (*r*, 0.40 to 0.80 or -0.40 to -0.80), or strong (*r*, 0.8 to 1.0 or -0.8 to -1.00); *p* values < 0.05 were considered statistically significant for paired t-tests. Excel Version 16.59 (Microsoft, Redmond, WA, USA) and STATA statistical software Version 16.0 (Stata Corp, College Station, TX, USA) were used for all calculations. A statistician was consulted, and analysis was performed accordingly.

## Results

### Population

The population of the study consisted of a total of 89 patients categorized as either normal (*n* = 31), muscular (*n* = 28), or overweight (*n* = 30). Descriptive and allometric parameters from the patient dataset in this study are shown in Table 2. Overall, average patient TBW, height, and BMI were 78 ± 18 kg, 175 ± 9 cm, and 25 ± 5 kg/m^2^, respectively. BMI and waist size varied between 14 to 40 kg/m^2^, and 64 to 131 cm, respectively.

**Table 2:** Descriptive statistics of demographic factors by body habitus presented as mean ± standard deviation with ranges

Demographic factors	Normal (*n* = 31)	Muscular (*n* = 28)	Obese (*n* = 30)
Age (years)	62.6 ± 9.3 (44–82)	34.3 ± 10.8 (19–56)	56.9 ± 13.7 (28–77)
Total body weight (kg)	67.9 ± 11.5 (38–88)	73.1 ± 15.1 (54–118)	93.9 ± 16.3 (70–136)
Height (cm)	174 ± 8.1 (160–187)	178 ± 10.7 (157–199)	174 ± 8.4 (160–194)
BMI (kg/m^2^)	22.2 ± 2.8 (14–26)	22.8 ± 2.8 (17–30)	31.0 ± 3.7 (25–40)
Waist size (cm)	84.2 ± 10.0 (64–100)	82.0 ± 10.2 (66–102)	107.9 ± 12.0 (88–131)
BFP (%)	25.8 ± 5.8 (15–41)	19.7 ± 5.2 (10–29)	36.2 ± 6.6 (23–48)
Lean body mass (kg)			
According to James	52.9 ± 9.0 (33–68)	57.3 ± 10.7 (41–85)	60.8 ± 10.5 (48–81)
According to Boer	53.2 ± 7.6 (39–66)	57.0 ± 9.7 (41–82)	60.9 ± 10.9 (47–86)
According to BIA	50.3 ± 9.4 (31–67)	58.6 ± 11.6 (38–86)	59.9 ± 12.2 (42–86)
Body surface area (m^2^)	1.8 ± 0.2 (1.3–2.1)	1.9 ± 0.2 (1.5–2.6)	2.1 ± 0.2 (1.8–2.7)

*BIA* Bioelectric impedance analyzer, *BMI* Body mass index, *BFP* Body fat percentage

### Calculated CM dose

**Fig. 2:**
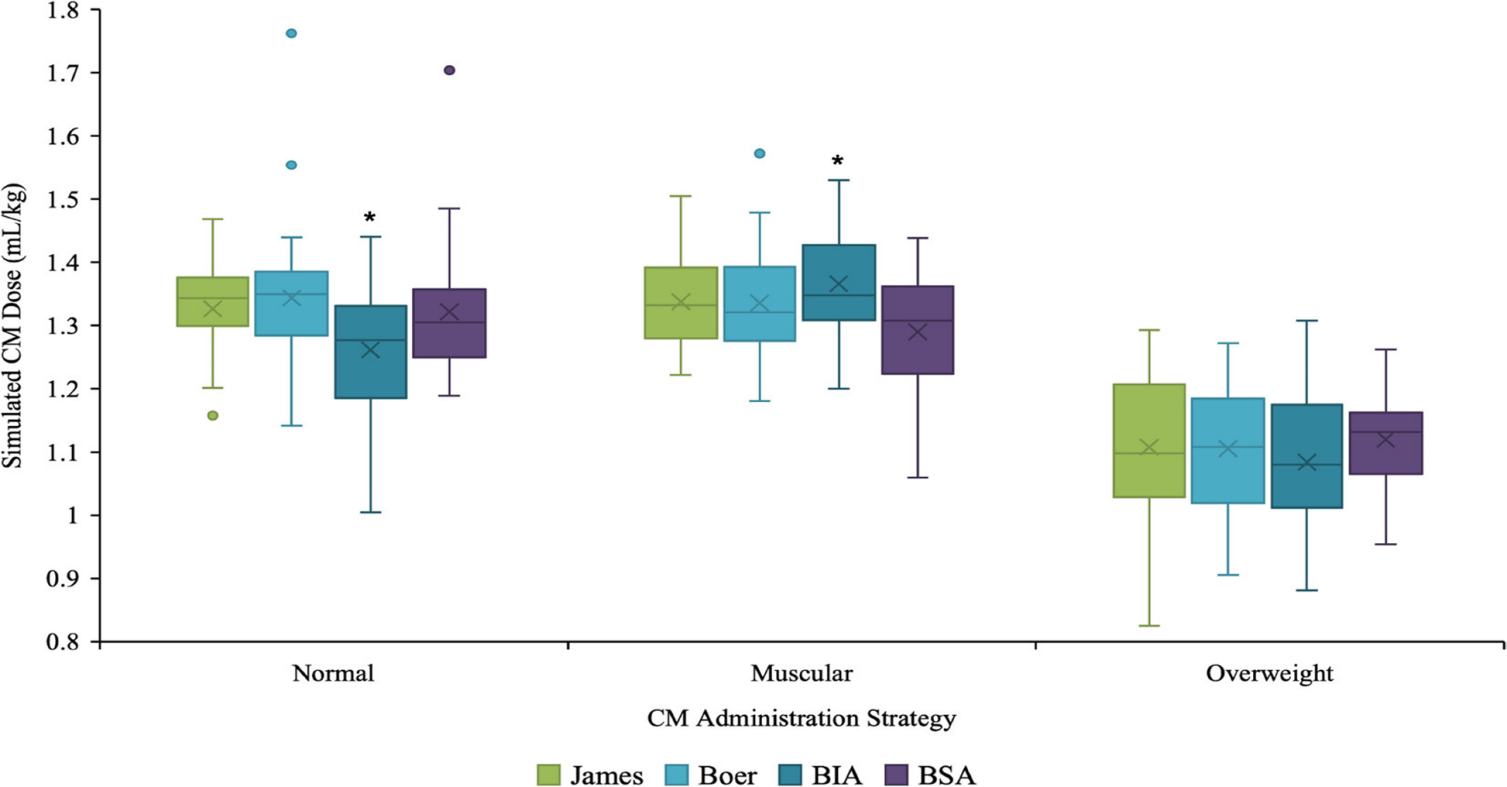
Box plot showing the calculated CM dose (mL/kg) for each CM strategy by body composition. Plot shows the mean ± standard deviation, the minimum and maximum values. The box represents 25th and 75th percentile values, and the whiskers indicate 1.5 times the interquartile range. Outlier values are plotted with dots. * = *p* < 0.05 between LBM BIA and other LBM strategies, including James *versus* BIA (*p* = 0.005) and Boer *versus* BIA (*p* = 0.004) for normal size patient group and BIA *versus* BSA (*p* = 0.003) for muscular patient group. *BIA*, Bioelectric impedance analysis; *BSA*, Body surface area; *CM*, Contrast medium; *LBM*, Lean body mass

Table 3 presents calculated CM doses according to normal, muscular, and overweight patient categories. Differences between mean CM dose (mL/kg) for each body composition using the included CM dose strategies in the present study are shown in Fig. 2. These differences were statistically significant (*p* < 0.050) for the three body compositions using TBW *versus* LBM and BSA strategies. Furthermore, differences in the normal group using LBM BIA *versus* LBM James (1.26 ± 0.10 mL/kg *versus* 1.33 ± 0.08 mL/kg; *p* = 0.005) and LBM Boer (1.26 ± 0.10 mL/kg *versus* 1.34 ± 0.12 mL/kg; *p* = 0.004) were statistically significant. In the muscular group a statistically significant (*p* = 0.003) difference was observed when comparing LBM BIA and BSA. Calculated CM dose was higher in muscular patients when using LBM BIA when compared to BSA as well as LBM James and LBM Boer (Table 3). Calculated CM doses (mL/kg) for all three-body compositions were, as expected, identical using the TBW strategy while a significant difference was observed with all other strategies included in this study (Table 3).

**Table 3:** Calculated contrast medium doses for each CM strategy in mL/kg and mL for the defined body compositions

Strategy	Body composition	Calculated CM dose Mean ± SD (range)	Calculated CM dose Mean ± SD (range)
		mL/kg	mL
TBW	Normal	1.20 ± 0.00 (1.20–1.20)	81 ± 13.8 (45–105)
	Muscular	1.20 ± 0.00 (1.20–1.20)	88 ± 18.2 (65–142)
	Overweight	1.20 ± 0.00 (1.20–1.20)	113 ± 19.5 (84–164)
LBM			
James	Normal	1.33 ± 0.08 (1.16–1.47)	90 ± 15.3 (56–115)
	Muscular	1.33 ± 0.07 (1.22–1.50)	97 ± 18.6 (69–144)
	Overweight	1.11 ± 0.11 (0.83–1.29)	103 ± 17.9 (82–138)
Boer	Normal	1.34 ± 0.12 (1.14–1.76)	90 ± 12.9 (67–112)
	Muscular	1.33 ± 0.09 (1.18–1.57)	97 ± 16.9 (68–139)
	Overweight	1.11 ± 0.09 (0.91–1.27)	103 ± 18.6 (80–146)
BIA	Normal	1.26 ± 0.10 (1.00–1.44)	86 ± 16.0 (53–113)
	Muscular	1.37 ± 0.09 (1.20–1.53)	100 ± 19.7 (65–147)
	Overweight	1.08 ± 0.11 (0.88–1.31)	102 ± 20.8 (72–146)
BSA	Normal	1.32 ± 0.11 (1.19–1.70)	89 ± 9.3 (65–104)
	Muscular	1.28 ± 0.09 (1.06–0.43)	93 ± 11.7 (76–125)
	Overweight	1.12 ± 0.08 (0.95–1.26)	104 ± 11.0 (88–130)

*BIA* Bioelectric impedance analyzer, *BSA* Body surface area, *CM* Contrast medium, *LBM* Lean body mass, *TBW* Total body weight

### Effect of patient demographics on calculated CM dose

Correlational analysis using Pearson’s correlation coefficient was performed to investigate the impact of different patient characteristics on CM dose for each body composition, according to TBW, LBM (James, Boer, and BIA) and BSA, as shown in Table 4. There were significant differences (*p* < 0.05 for all) in correlation between calculated CM doses and several demographic factors as shown in Table 4. The table also shows a slightly stronger negative correlation for BFP using BIA than the other LBM calculations. These differences had a statistically significant impact (*p* < 0.05) for normal and overweight groups using LBM BIA (*p* = 0.03 and *p* = 0.00, respectively) and only for the overweight group using LBM James and Boer (*p* = 0.01 and *p* = 0.03 respectively).

**Table 4:** Pearson’s correlation coefficients (*r*) and *p* values of demographic factors and calculated contrast medium dose

Strategy	Demographics	Normal	Muscular	Overweight
TBW	Total body weight (kg)	1.00*	1.00*	1.00*
	Height (cm)	0.71*	0.77*	0.74*
	BMI (kg/m^2^)	0.84*	0.84*	0.83*
	Waist circumference (cm)	0.74*	0.89*	0.81*
	BFP (%)	0.06	0.16	-0.01
LBM	Total body weight (kg)	0.95*	0.96*	0.81*
James	Height (cm)	0.83*	0.88*	0.94*
	BMI (kg/m^2^)	0.66*	0.70*	0.40*
	Waist circumference (cm)	0.76*	0.88*	0.74*
	BFP (%)	-0.19	-0.06	-0.49*
LBM	Total body weight (kg)	0.90*	0.95*	0.88*
Boer	Height (cm)	0.90*	0.91*	0.92*
	BMI (kg/m^2^)	0.54*	0.65*	0.51*
	Waist circumference (cm)	0.70*	0.86*	0.78*
	BFP (%)	-0.26	-0.09	-0.40*
LBM	Total body weight (kg)	0.91*	0.95*	0.86*
BIA	Height (cm)	0.80*	0.85*	0.86*
	BMI (kg/m^2^)	0.63*	0.71*	0.53*
	Waist circumference (cm)	0.69*	0.85*	0.73*
	BFP (%)	-0.36*	-0.14	-0.51*
BSA	Total body weight (kg)	0.91*	0.95*	0.86*
	Height (cm)	0.80*	0.86*	0.84*
	BMI (kg/m^2^)	0.75*	0.75*	0.73*
	Waist circumference (cm)	0.73*	0.88*	0.79*
	BFP (%)	-0.02	0.07	-0.10

Bold highlights Pearson correlations between calculated contrast medium dose and demographics found to be statistically significant. *BFP* Body fat percentage, *BIA* Bioelectric impedance analyzer, *BMI* Body mass index, *BSA* Body surface area, *LBM* Lean body mass, *TBW* Total body weight

^*^Statistical significance between the calculated CM dose and demographic factors (*p* < 0.05)

## Discussion

This study aimed to evaluate which method of CM dose calculation (TBW, three LBM CM dose calculation methods, and BSA) would be the most appropriate approach to be used in a larger future prospective study evaluating contrast enhanced thoracic CT examinations. The evaluation was performed based on CM dose calculation according to several methods and analyzed the impact of demographic factors and body habitus in an existing dataset, including three different body compositions.

Calculated CM doses for each body habitus group produced similar results using the James, Boer, and BSA methods (Tables 3 and 4). Among LBM strategies, the calculated CM dose was lower in the normal group (1.26 mL/kg) and overweight group (1.08 mL/kg) and higher in muscular group (1.37 mL/kg) using BIA compared to James and Boer as shown in Table 3, demonstrating BIA had the largest variation in CM dose according to body composition. When compared to the normal group and using BIA, the muscular group received higher CM dose by 11% (0.11 mL/kg) (*p* = 0.00) and lower than 14% (0.18 mL/kg) for overweight participants (*p* = 0.00). Clinically, using the BIA method, a 90-kg patient would receive 113 mL, 123 mL, and 97 mL of CM having a normal, muscular, or overweight body habitus, respectively, showing a higher variability between the three body habitus groups when compared to James (120, 120, and 100 mL) and Boer (121, 120, and 100 mL) methods as well as TBW (90 mL for all three groups). This greater variation in estimated CM dose using BIA is due to the larger variation in BFP% between the body composition categories.

The use of personalized CM doses has been reported with varied results concerning both CM dose and CM enhancement. While some studies have demonstrated minimal variation between CM dose calculations [5, 22], others have demonstrated higher variations [8, 20]. Zanardo et al. [22] found comparable image quality using TBW and LBM. A limitation of this study, however, was that it did not include patients with a varied body habitus. When a more diverse population is included, variation in CM enhancement has been reported [7, 8]. Differences in CM enhancement have also been reported using different LBM strategies [6, 11]. Rengo et al. [6] found that measurements of LBM using BIA were optimal for tailoring CM dose for hepatic CT exams. As the BIA method better reflects the variations in patient body habitus, it accounts for an increase in CM needed for highly vascular parenchymal organs within muscular patients, and a decrease in CM required to diffuse adipose tissue in overweight patients [1, 6, 8]. Clinically, due to this higher vascularization [10], muscles are perfused with proportionally more blood and consequently more contrast medium than adipose tissue. Therefore, this strategy may better account for this difference, resulting in a more homogenous enhancement regardless of body composition. It is also known that LBM strategies correct for high BFP by correlating with extracellular fluid volumes [4, 6, 12]. This is confirmed in our results that show a higher BFP for normal and overweight groups, as reported in Table 2. Another benefit of using BIA is that it accounts for only weight and BFP [12] compared to James and Boer that include height, weight and gender [11].

It is important to note that no contrast enhancement was analyzed and included in our study, as the CM calculations and analysis were performed retrospectively. Contrast enhancement will be included in the future larger prospective clinical study.

The correlational analysis of patient demographic factors produced some expected results. The ratio between weight and CM dose for the TBW approach was 1.0, which was predictable given that weight was the determinant factor in the strategy. Similarly, the strong correlation between patient weight and height with CM dose when the James, Boer, and BSA methods were used was predictable, as these factors were directly used in the calculations presented in Table 1. While the BIA formula does not utilize patient height, there remained a strong correlation between height and CM dose for this strategy, independent of body habitus. This finding was intuitively expected due to the proportional relationship between weight and height (*i.e.*, taller people generally weigh more than shorter people) [8]. Of the LBM methods, the BIA method had the strongest statistically significant inverse relationship between BFP and CM dose, as expected from the equation of LBM BIA. Therefore, patients with a higher BFP would receive lower CM dose. This finding supports using BIA, as it does not overestimate CM doses for overweight patients with low metabolic mass. The BIA method also had weaker correlations than other included strategies between both BMI and waist size to CM dose. This is ideal given that BMI is a poor estimate of total body fat content in muscular people with high BMIs due to their muscle density [8]. In this sense, the James formula is shown to be less accurate in obese patients with BMI ≥ 37 and ≥ 43 kg/m^2^ for female and male, respectively, as estimates reach a level where the estimated LBM decreases with increasing BMI [11, 23]. However, the current study included only one patient with BMI 39.5 kg/m^2^ and is therefore limited in ability to investigate the impact of using the James or Boer methods. Moreover, formulas cannot accurately substitute for quantitative imaging or measurements [24]. This is especially important when analysis focuses on measurements of muscle mass or changes in body composition. Chamchod et al. [24] concluded that CT based LBM assessments were more reliable than formulas in cancer patients at risk for illness and therapy associated changes in body composition. With regard to CM enhancement and increased vascularization in muscle tissue, the use of BIA could be of benefit to discriminate high muscle mass subjects from obese ones, especially in patients with comparable body size measures like BMI.

Our study has some limitations. Our calculations were based on predefined CM doses from a series of patients who received the standard CM dose in clinical practice. Also, this study used only theoretical calculations which have not yet been supported by image analysis to confirm our results. However, we expect that the prospective ongoing study confirms our hypothesis. Thirdly, the use of BIA analyzer could carry some known inaccuracy and reproducibility issues; however, the scientifically validated analyzer was used with a standardized procedure and the technique itself has been validated in previous studies [25–27]. Lastly, a subjective analysis of only 16 muscular patients older than 30 years old might result in less accurate assignment of body composition category, however, the technologists involved in the patient recruitment were trained carefully, and thus any negative impact on patient categorization would be limited. Moreover, BFP results suggest the categorization is adequate.

In conclusion, our estimations of CM dose based on various CM dose calculations showed that there were significant differences between TBW and all the included more compound CM dosing strategies. The BIA method demonstrated a closer correlation to various patient demographics included in the current study and determined greater differences, especially in muscular and overweight patients. To fully investigate the clinical impact of different dose calculation methods on various body compositions, further studies need to be carried out comparing variations in contrast dosage depending on body habitus, assessing both contrast enhancement and image quality.

## Data Availability

The dataset is available from the corresponding author on reasonable request.

## Abbreviations

BFP: Body fat percentage
BIA: Bioelectric impedance analysis
BMI: Body mass index
BSA: Body surface area
CM: Contrast medium
CT: Computed tomography
TBW: Total body weight
